# Extracellular electron uptake from a cathode by the lactic acid bacterium *Lactiplantibacillus plantarum*

**DOI:** 10.3389/fmicb.2023.1298023

**Published:** 2023-11-23

**Authors:** Sara Tejedor-Sanz, Siliang Li, Biki Bapi Kundu, Caroline M. Ajo-Franklin

**Affiliations:** ^1^Department of BioSciences, Rice University, Houston, TX, United States; ^2^Biological Nanostructures Facility, The Molecular Foundry, Lawrence Berkeley National Laboratory, Berkeley, CA, United States; ^3^PhD Program in Systems, Synthetic, and Physical Biology, Rice University, Houston, TX, United States; ^4^Department of Bioengineering, Rice University, Houston, TX, United States; ^5^Department of Chemical and Biomolecular Engineering, Rice University, Houston, TX, United States

**Keywords:** lactic acid bacteria, extracellular electron transfer, extracellular electron uptake, biocathode, electro-fermentation, bioelectrosynthesis

## Abstract

A subset of microorganisms that perform respiration can endogenously utilize insoluble electron donors, such as Fe(II) or a cathode, in a process called extracellular electron transfer (EET). However, it is unknown whether similar endogenous EET can be performed by primarily fermentative species like lactic acid bacteria. We report for the first time electron uptake from a cathode by *Lactiplantibacillus plantarum*, a primarily fermentative bacteria found in the gut of mammals and in fermented foods. *L. plantarum* consumed electrons from a cathode and coupled this oxidation to the reduction of both an endogenous organic (pyruvate) and an exogenous inorganic electron acceptor (nitrate). This electron uptake from a cathode reroutes glucose fermentation toward lactate degradation and provides cells with a higher viability upon sugar exhaustion. Moreover, the associated genes and cofactors indicate that this activity is mechanistically different from that one employed by lactic acid bacteria to reduce an anode and to perform respiration. Our results expand our knowledge of the diversity of electroactive species and of the metabolic and bioenergetic strategies used by lactic acid bacteria.

## Introduction

Bacteria oxidize a variety of electron donors and harness the released energy to generate ATP. The electron donors can either diffuse across the cell envelope into the intracellular space, e.g., hydrogen in hydrogen-oxidizing bacteria (Aragno and Schlegel, 1981), or remain extracellular, e.g., Fe(II) for Fe-oxidizing bacteria (Emerson et al., 2010). Whether the electron donor is intracellular or extracellular determines the electron transfer proteins and pathways used by bacteria to access, interact, and gain energy from redox reactions. When the electron donor is extracellular, this process is called extracellular electron uptake.

Understanding the naturally-occurring extracellular electron transfer (EET) pathways that enable bacteria to perform extracellular electron uptake has become an area of great fundamental and applied interest (Tremblay et al., 2017; Su and Ajo-Franklin, 2019; Gupta et al., 2020). Identifying the mechanism of electron transfer from solid electron donors, like Fe(II)/Mn (II)-forms (Gupta et al., 2019; Keffer et al., 2021) or elemental S (Cron et al., 2019), to microbes would further our knowledge of biogeochemical cycles and ecological interactions among microbial communities in specific niches. In some cases, these microbes can also uptake electrons from a cathode poised at an equivalent redox potential in place of their physiological electron donor (Rowe et al., 2015; Gupta et al., 2020). Studying this phenomenon establishes the basis for developing biotechnological applications of cathodic driven microbial reactions in bioremediation (Tejedor-Sanz et al., 2020), biosensing (Zhou et al., 2017) and bioelectrosynthesis (Nevin et al., 2011; Harnisch and Holtmann, 2019).

Recent work has expanded our knowledge of microorganisms with an endogenous ability to perform extracellular electron uptake from cathodes (cathodic EET). These include acetogens and methanogens (Villano et al., 2010), sulfur and sulfate-reducing bacteria (Rowe et al., 2015; Agostino and Rosenbaum, 2018), denitrifiers (Tejedor-Sanz et al., 2016), photosynthetic and non-photosynthetic Fe^2+^-oxidizing bacteria (McAllister et al., 2019; Gupta et al., 2020). All these microorganisms reduce external electron acceptors to conserve energy and perform respiration. However, extracellular electron uptake is largely unexplored across primarily fermentative bacteria, that is, species that mainly reduce endogenous electron acceptors to conserve energy. Several fermentative species can uptake electrons from a cathode when an artificial redox compound is added or when very low cathode potentials are applied (Kracke et al., 2016; Zhang et al., 2021), however, they cannot endogenously uptake electrons from extracellular donors. To date, only the Gram-positive and primarily fermentative bacterium *Clostridium pasteurianum* (Choi et al., 2014) has been described to endogenously uptake electrons from a cathode. Interestingly, electron uptake in this species affects its intracellular redox balance and triggers a metabolic shift toward NADH-consuming routes, impacting how *C. pasteurianum* conserves energy. These observations show that at least one fermentative species can natively uptake electrons from a cathode, suggesting that other yet-unidentified primarily fermentative species may also possess a similar endogenous capacity.

To identify other fermentative species that are able to uptake electrons from a cathode, we must look in different ecological niches in which this phenomenon was never explored before. We had recently characterized EET pathways involving reduction of anodes and insoluble iron in multiple LAB species (Light et al., 2018; Tejedor-Sanz et al., 2022), primarily fermentative species found in the mammalian gut and fermented foods. Interestingly, this EET ability allowed the LAB *Lactiplantibacillus plantarum* to enhance fermentation flux and yield using a hybrid energy acquisition mechanism that blends features of fermentation and respiration. In addition to reducing iron or anodes, *L. plantarum* has other electron transfer pathways that use external electron acceptors like oxygen and nitrate (Brooijmans R. J. W. et al., 2009). These observations led us to hypothesize that this species could also possess an ability to uptake electrons from a cathode. In this study we explore electron uptake in *L. plantarum*, the mechanisms involved, and its impact on cell metabolism.

## Results

### *Lactiplantibacillus plantarum* consumes current when supplied with nitrate as an electron acceptor

Some microorganisms that endogenously uptake electrons from extracellular electron donors can also uptake electrons from cathode poised at an equivalent redox potential, coupling this electron uptake to reduction of an electron acceptor (Rowe et al., 2015). To determine if *L. plantarum* can uptake electrons from a cathode, we first tested whether it could consume current under different cathode potentials with an external physiological electron acceptor (nitrate) present (Brooijmans R. J. W. et al., 2009) in a bioelecrochemical reactor (Supplementary Figures S1A,B). To promote growth without usurping the role of the cathode as the electron donor, we supplied 5 mM glucose as a carbon source since under these conditions *L. plantarum* minimally utilizes the glucose to reduce nitrate (Brooijmans R. J. W. et al., 2009) (if the electron transport chain is reconstituted). We detected current consumption in a minimal medium (Aumiller et al., 2023) by *L. plantarum* under all the potentials tested (−0.15, −0.25, −0.40 and − 0.60 V), with the highest current at −0.60 V (all potentials are reported vs. Ag/AgCl_KCl sat_) (Supplementarys Figure S1A,B). No current consumption was observed with heat killed cells under identical conditions (Supplementary Figure S1C), indicating that electron uptake required metabolically active cells. We also observed a higher and earlier current consumption when glucose was supplied (Supplementary Figure S1D), suggesting that cells could maintain a higher metabolic activity that resulted in a greater EET from the cathode. Because of all these observations, we chose to use a potential of −0.6 V and to provide 5 mM glucose to the medium for further experiments to maximize EET from the cathode.

**Figure 1:**
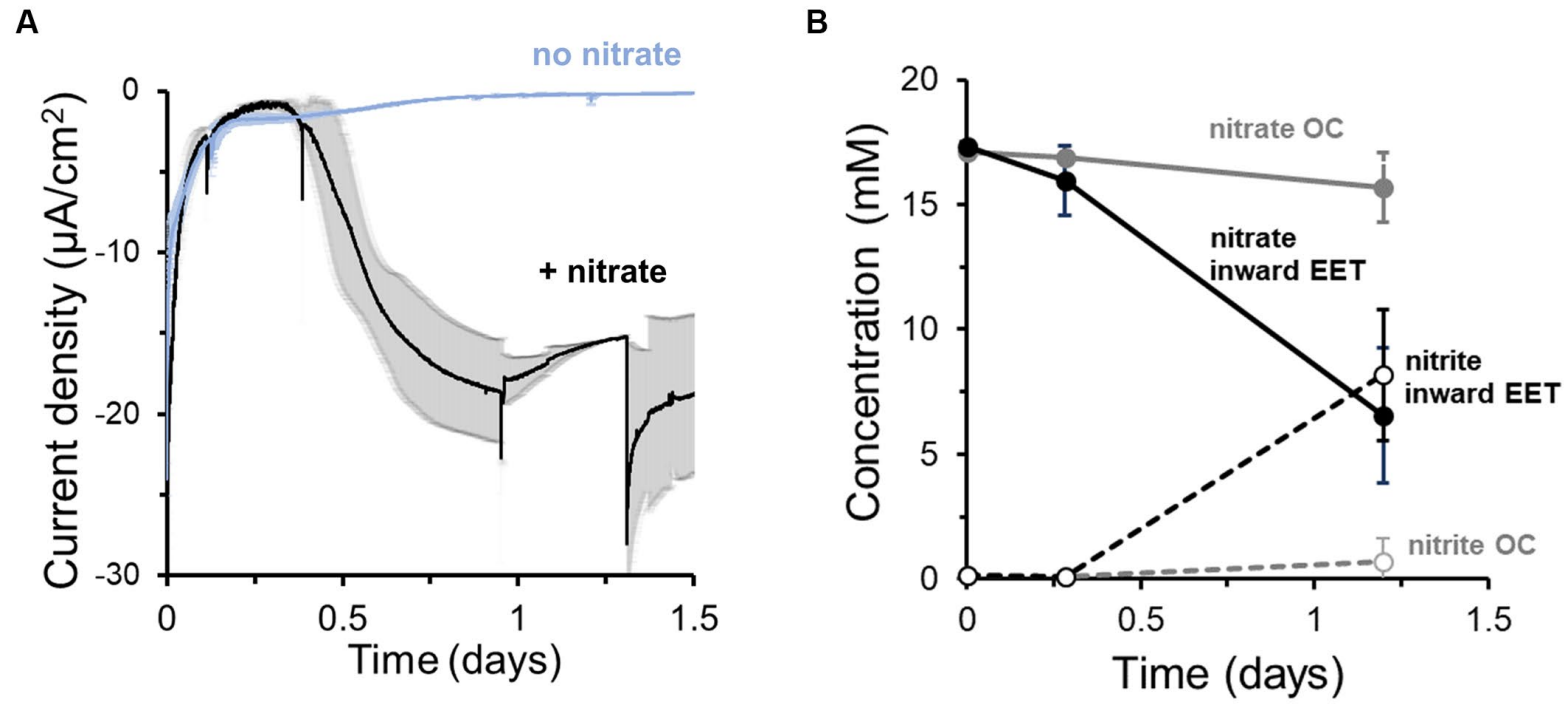
*Lactiplantibacillus plantarum* endogenous extracellular electron uptake capacity from cathodes to reduce nitrate. **(A)** Current density over time in the presence and absence of nitrate as electron acceptor, showing that nitrate presence triggers current consumption. **(B)** Nitrate consumption (continuous lines) and nitrite production (dotted lines) over time in the presence of a polarized cathode (EET) (closed circles) and with an electrode under open circuit (OC) condition. For the experiment, nitrate and glucose (5 mM) were already present in the medium at *t* = 0, ΔE_cathode_ = −600 mV_Ag/AgCl, sat. KCl_ and the error bars indicate the standard deviation from triplicate bioelectrochemical reactors.

To probe whether this electron uptake was coupled to the reduction of an electron acceptor, we next monitored current in the presence and absence of nitrate (Figure 1A). Approximately 18 h after inoculation of cells with nitrate, a maximum current consumption of −20 ± 5 μA/cm^2^ at −0.60 V (Figure 1A) was observed. However, no significant current consumption occurred in the absence of nitrate (Figure 1A), indicating that the extracellular electron uptake required an external electron acceptor. The electron uptake also coincided with the reduction of nitrate to nitrite (Figure 1B). We compared these data to nitrate reduction by *L. plantarum* under open circuit (OC) conditions, in which the cathode is not electrically connected to the anode and thus electrons from electrical current cannot be supplied. Under OC conditions, *L. plantarum* reduced significantly less nitrate to nitrite (1.19 ± 1.16 mM vs. 8.98 ± 2.29 mM per day, *p* < 0.050) (Figure 1B). Using differential pulse voltammetry (Supplementary Figure S1E), we identified a redox active center at −0.52 V (pH = 6.3, vs. Ag/AgCl sat. KCl) which was not observed when nitrate or cells were not present in the medium. Thus, this result confirms the existence of a redox process associated with the bioelectrochemical reduction of nitrate or nitrite, similar to other observations (Pous et al., 2014). Further experiments indicated that nitrite could be biotically and abiotically reduced in the reactors at −0.6 V (Supplementary Figure S2A). The addition of nitrite in a bioelectrochemical reactor with heat killed cells showed a significant current consumption, which was slightly breater when viable cells were present (Supplementary Figure S2A) (ΔE = −0.6 V). Moreover, 1.3 mM of nitrite was consumed over 3 days in the presence of heat killed cells while 1.6 mM was consumed when viable cells were added (Supplementary Figure S2B). To understand the use of the cathode by *L. plantarum* to reduce nitrite, we minimized the abiotic electrochemical signal by reducing the cathode potential to −0.25 V and −0.4 V. Under these cathode potentials, the addition of nitrite led to a minimal current consumption, and adding viable cells did not vary current consumption levels (Supplementary Figure S2C), while significant nitrite was reduced (Supplementary Figure S2D). Thus, the nitrite biological reduction was mainly occurring without the mediation of the cathode (>91%) (see SI for electron balances). This indicated that *L. plantarum* predominately uses electrons from a cathode to drive the reduction of nitrate to nitrite, but a separate pool of electron donors is used by *L. plantarum* to reduce nitrite.

Through its fermentative pathways, *L. plantarum* produces organic molecules, e.g., acetate, that can also serve as electron donors. To establish to what degree the cathode vs. endogenous molecules served as the electron donor for *L. plantarum* to reduce nitrate, we quantified the electron uptake needed for nitrate reduction. We first calculated the electrons consumed from the cathode from the current consumption data and compared this value to the number of electrons theoretically needed to reduce the consumed nitrate to nitrite in the bioreactor medium (Figure 1B). Significantly more electrons were used to reduce nitrate than electrons supplied by the cathode: only 6.9 ± 1.6% of the electrons needed for nitrate reduction were supplied by the cathode (i.e., coulombic efficiency). This difference indicates that additional electron donors, such as an organic fermentation intermediates, may be utilized by source for *L. plantarum* to reduce nitrate.

### EET from a cathode in *Lactiplantibacillus plantarum* induces a metabolic shift that enhances cell viability

**Figure 2:**
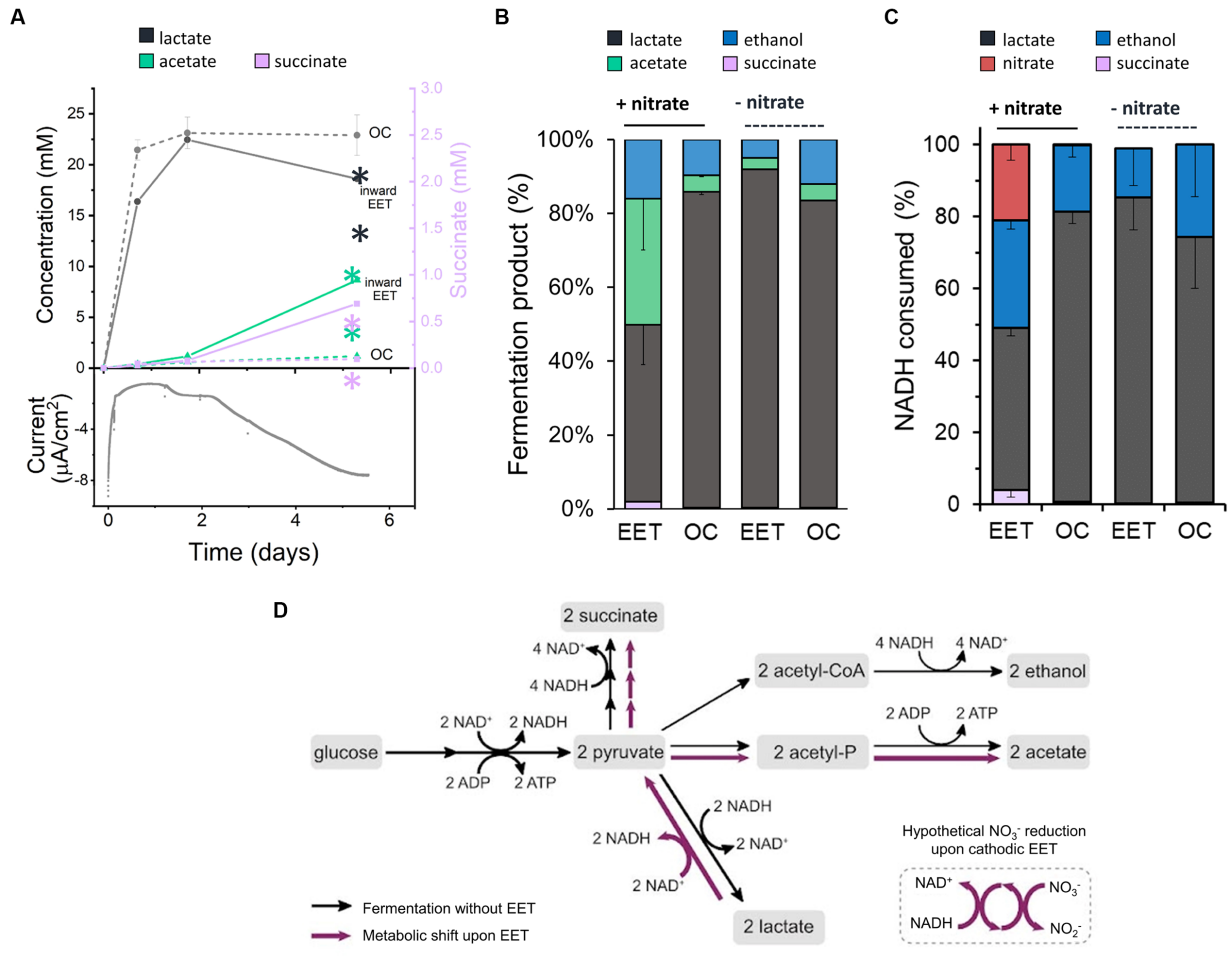
Impact of electron uptake on fermentative metabolism. **(A)** Evolution of metabolites under EET (continuous lines) and OC conditions (dotted lines) in the presence of both nitrate and glucose. Current consumption is shown for 1 reactor representing the EET condition tested in triplicate. Lactate (grey) and acetate (green) are represented on the left-Y axis of the graph, while succinate (purple) on the right-Y axis. **(B)** Metabolites produced under EET and OC conditions in the presence and absence of nitrate by day 5. **(C)** Estimated fractions of NADH consumed over global NADH consumed from nitrate reduction and fermentation pathways leading to lactate, ethanol and succinate production under EET vs. OC conditions in the presence and absence of nitrate by day 5. **(D)** Proposed metabolic shift of glucose fermentation under EET vs. OC conditions. Black arrows indicate the metabolic pathways under OC and under EET conditions before electron uptake occurs. Purple arrows indicate the proposed metabolic pathways that are stimulated when electron uptake occurs. For all experiments, nitrate and glucose (10 mM) were already present in the medium at *t* = 0, ΔE_cathode_ = −600 mV_Ag/AgCl,sat.KCl_ and the error bars indicate the standard deviation from triplicate bioelectrochemical reactors. Significant differences were determined by one-way ANOVA with Dunn-Sidak post-hoc test (*n* = 3), ** *p* ≤ 0.01.

When *L. plantarum* donates electrons to an extracellular anode, it conserves energy more rapidly and increases its fermentative flux (Tejedor-Sanz et al., 2022). To probe the impact of the electron uptake on energy conservation and metabolism, we measured metabolites of *L. plantarum* under OC and cathodic EET conditions from glucose fermentation, employing this time 10 mM of sugar to facilitate the analyses of metabolites. Under EET and OC conditions, *L. plantarum* predominantly produced the end products of fermentation lactate, ethanol, acetate and succinate (Figures 2A,B). Formate (<0.02 mM) and pyruvate (<0.06 mM) were detected in all bioreactors at trace levels, and no acetoin or 2,3-butanediol were detected at any time. By day 5, these fermentation products contained >98% of the initial carbon supplied as glucose, indicating these measurements provide a complete view of *L. plantarum* carbon metabolism under these conditions.

Interestingly, the fermentation process was affected by electron uptake in *L. plantarum*. Over the first 1.7 days, fermentation products were similar under EET and OC conditions (Figure 2A). Glucose was fully consumed in all reactors after 24 h (Supplementary Figure S3A), lactate was the main fermentation product (22.5 ± 0.1 mM), and the nitrate concentration was unchanged (Supplementary Figure S3B). After day 1.75, however, there was a gradual increase in current consumption accompanied by nitrate consumption (238 μmol day ^−1^ vs. 5.5 μmol day ^−1^ under OC conditions) (Figure 2A; Supplementary Figure S3B). Moreover, we observed a significant increase in production of acetate and succinate and an increased consumption of lactate under EET conditions. For instance, lactate consumption rates were of 35.8 μmol day ^−1^ under EET conditions and of 9.8 μmol day ^−1^ under OC conditions. Thus, electron uptake and nitrate reduction coincided with a shift of *L. plantarum* metabolism toward a more heterogeneous distribution of end products (Figure 2B, 46% lactate, 38% acetate, 15% ethanol and 2.3% succinate) than under OC conditions, in which lactate remained the dominant product. This strongly suggests that, after exhausting glucose, *L. plantarum* utilizes the cathode as an electron donor and nitrate as an electron acceptor, allowing lactate to be sequentially oxidized to pyruvate then acetate (Figure 2D). The absence of nitrate or the polarized cathode did not induce the metabolic shift, probing that this phenomenon is associated with electron uptake to reduce nitrate (Figure 2B). We also observed this shift in the spectrum of end-metabolites when we repeated the test using the lower concentration of glucose (5 mM) we employed in previous assays (Supplementary Figures S3C,D, S5). In fact, even higher relative levels of acetate (41%) were detected and lower levels of lactate (29%), while we observed an enhanced current consumption in the bioelectrochemical reactors. This suggests that the higher the electron uptake, the more pronounced the metabolic shift is.

Our results indicate a complex electron flow during extracellular electron uptake: the cathode and an endogenous fermentation product (lactate) serve as electron donors, while exogenous nitrate acts as an electron acceptor. To understand the impact of this electron flow on the intracellular redox state of *L. plantarum*, we analyzed how the reducing equivalents generated from glycolysis, i.e., NADH, and the electrons from the cathode were used. For this analysis, we estimated the NADH regenerated via fermentation and via the reduction of nitrate using the measured concentrations of end-fermentation products and nitrate (see Methods section for methodology). Under OC conditions, we estimated that a 81%, a 18% and a 0.7% of all the NADH was regenerated via lactate ethanol and succinate production, respectively (Figure 2C), in agreement with prior observations of homofermentation (Ferain et al., 1996). However, when cells consumed electrons from the cathode (EET + nitrate), NADH regeneration via lactate was reduced to 45, 30% via ethanol, 21% of the via the reduction of nitrate to nitrite and a 4% via succinate (Figure 2C). Thus, electron uptake by *L. plantarum* changes the strategy that it uses to balance its intracellular redox state.

**Figure 3:**
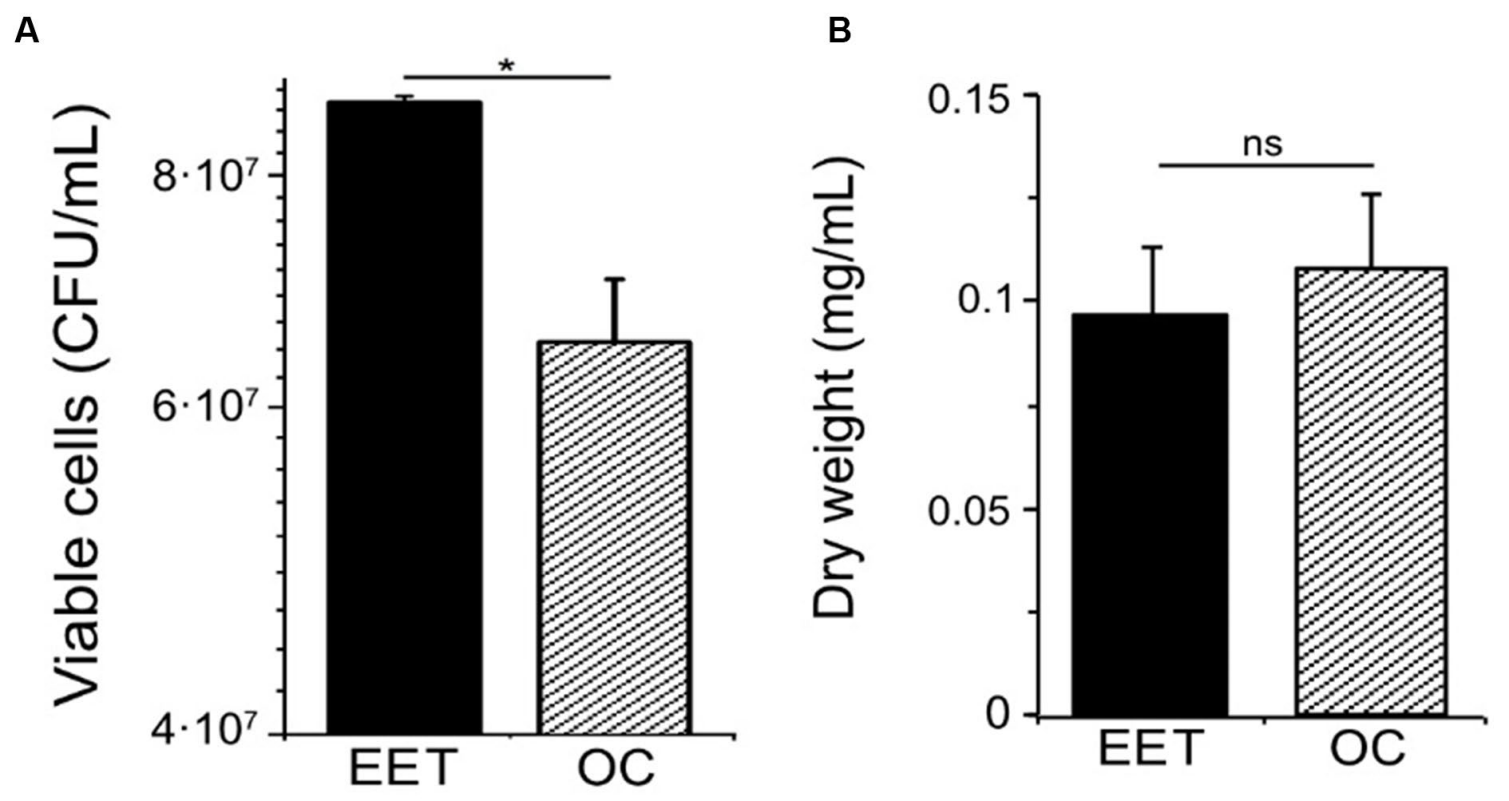
Impact of electron uptake impact on cell viability and final biomass in *L. plantarum*. **(A)** Cell viability at day 2 and **(B)** final biomass (dry weight) (day ∼5) for both electron uptake (EET) and OC conditions. For all experiments, nitrate (10 mM) and glucose (5 mM) were already present in the medium when the electrodes were polarized (ΔE_cathode_ = −600 mV_Ag/AgCl, sat. KCl_), and the error bars indicate the standard deviation from three replicates. Significant differences in iron reduction were determined by one-way ANOVA with Dunn-Sidak post-hoc test (*n* = 3), * *p* ≤ 0.05.

Extracellular electron transfer can affect catabolic processes and how they are coupled to anabolic processes, including cell growth. To examine how electron uptake impacted *L. plantarum* anabolism, we measured cell viability and biomass under EET and OC conditions. Even though we did not observe visible cell attachment, for quantifying growth we employed a graphite rod, an electrode with a smoother surface than graphite felt, to reduce cell attachment. When *L. plantarum* cultures produced the maximal current density (day 2), they contained a higher number of viable cells compared to OC conditions (Figure 3A). However, maximal current consumption (Supplementary Figure S4A) coincided with the entry of *L. plantarum* into the stationary. This observation suggests that when *L. plantarum* anabolism slows due to substrate depletion (glucose), electron uptake increases. Further supporting this observation, there were no significant differences in the cell density over time (Supplementary Figure S4A) or final biomass (Figure 3B) between OC and EET conditions, both in the presence and absence of nitrate (Supplementary Figure S4B). Thus, electron uptake coupled to nitrate reduction increases cell viability, but does not increase final biomass. We hypothesize that metabolic shift observed toward acetate production when electron uptake occurs may generate extra ATP, providing the cells with the additional energy needed for survival in the stationary phase and under glucose depravation. However, it may not be enough to support significant, if any, additional growth.

### Cathodic current consumption involves a different mechanism than respiratory and EET chains

**Figure 4:**
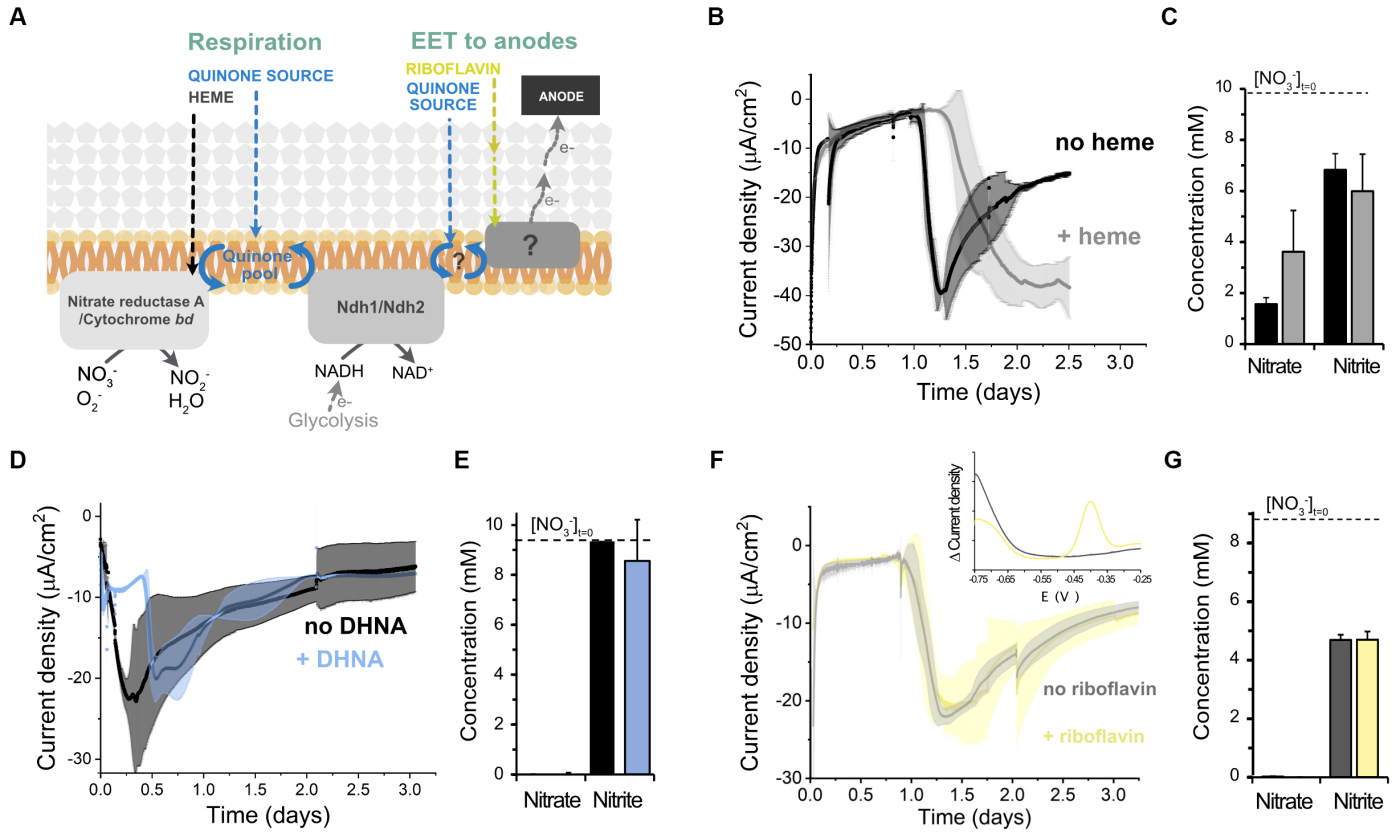
Effect of different electron transport cofactors on *L. plantarum* electron uptake. **(A)** Schematic of the known electron transport chains in *L. plantarum* associated with aerobic respiration, anaerobic respiration, and EET to anodes, showing the cofactors required for the activation of each electron transport chain. **(B,C)** Effect of heme supplementation on current density consumption **(B)** and nitrate and nitrite levels **(C)** under cathodic EET conditions. **(D,E)** Effect of the supplementation of the quinone synthesis precursor DHNA on current density consumption **(D)** and nitrate and nitrite levels **(E)**. **(F,G)** Effect of riboflavin on current density consumption **(F)** and nitrate and nitrite levels **(G)**. The inset in **(F)** shows the voltammogram obtained from a differential pulse voltammetry assay that confirmed the absence of flavins in the media (peak centered at ~ −0.4 V). For all experiments, nitrate (10 mM) and glucose (5 mM) were already present in the medium when the electrodes were polarized (ΔE_cathode_ = −600 mV_Ag/AgCl, sat. KCl_), and the error bars indicate the standard deviation from three replicates.

We next explored the molecular mechanisms supporting electron uptake. We studied whether cathodic EET requires molecules that activate aerobic and anaerobic respiration, heme and 1,4-dihydroxy-2-naphthoic acid (DHNA), respectively (Brooijmans R. et al., 2009), by adding these molecules exogenously and examining current consumption and nitrate reduction (Figure 4A). Heme addition did not affect the magnitude of the current consumed, but delayed the start of electron uptake by cells (Figure 4B). Heme supplementation did not affect nitrate reduction either (Figure 4C). Similarly, the addition of DHNA (Figures 4D,E) and omission of riboflavin (Figures 4F,G) did not impact current consumption nor nitrate reduction. Differential pulse voltammetry on the cathode of the riboflavin-free bioreactors also probed that residual riboflavin was not acting as a freely-diffusing electron shuttle since the characteristic redox peak of flavins at −0.4 V was absent from the cathodic chamber (inset of Figure 4E). This result is in accordance with the inability of *L. plantarum* to synthesize riboflavin (Brooijmans R. J. W. et al., 2009). Thus, the extracellular electron uptake machinery does not require any of the external cofactors, i.e., heme, quinone-precursors, or free riboflavin, necessary to activate the other known electron transfer chains in *L. plantarum*, other LAB and EET in other species (Wu et al., 2014; Mevers et al., 2019).

Those results indicate that *L. plantarum* uses electrons from a cathode directly, or via a mediated approach by either using other molecules present in the culturing medium acting as electron shuttles or by self-synthesizing them. We found that cysteine, a redox active compound that can behave as an electron shuttle between other electroactive species and minerals (Doong and Schink, 2002; Liu et al., 2014) was present at 0.8 mM in the medium used. Removing cysteine from the medium resulted in similar current consumption levels by *L. plantarum* than when cysteine was added to the medium (Supplementary Figures S5A,B). Thus, cysteine is not a mediator for cathodic electron uptake. Since no other compounds present in the CDM are known to act as electron shuttles, we posit that cells were performing direct EET with the cathode or were using a yet unidentified self-synthesized shuttle molecule or detached protein.

We next sought to further understand the mechanism involved by exploring the role of different proteins in EET in *L. plantarum*. Lactic acid bacteria, including *L. plantarum*, possess a flavin-based extracellular electron transfer (FLEET) locus (Supplementary Figure S6A) that is associated with an ability to perform EET with insoluble electron acceptors (Light et al., 2018; Tejedor-Sanz et al., 2022; Tolar et al., 2023). Because certain proteins in other bacterial species can be used for both interacting with insoluble donors and acceptors (Xie et al., 2021), we interrogated the role of certain genetic elements within the FLEET locus in the cathodic EET chain. Since DHNA is not required for electron uptake, we reasoned that DmkB (heptaprenyl diphosphate synthase complex II), an enzyme which catalyzes terminal steps in the production of menaquinone-7 from DHNA, is unlikely to be required for it. Instead, we targeted Ndh2 (the type II NADH-dehydrogenase), PplA, EetA, and EetB proteins, since they have a role in reducing insoluble electron acceptors in this species (Tejedor-Sanz et al., 2022; Tolar et al., 2023). Electron uptake, quantified as total charge consumed from the cathode (Supplementary Figure S6B), and nitrate reduction by Δ*ndh2*, Δ*pplA*, *and* Δ*eetA/B* strains (Supplementary Figures S6C,D) were similar to wild-type. These results indicate PplA, Ndh2 and the proteins EetA and EetB are not components of the cathodic EET chain. Because of this finding and our previous result showing that riboflavin is not required for electron uptake, we posit that FmnA and FmnB, proteins involved in the flavinilyzation of PplA (Light et al., 2018) and with their respective coding-genes within the FLEET locus, are unlikely to be essential elements in the cathodic EET chain. Overall, this analysis indicates that FLEET proteins are not redox enzymes mediating the electron uptake capacity in *L. plantarum*, confirming that the cathodic and anodic EET mechanisms are genetically different.

**Figure 5:**
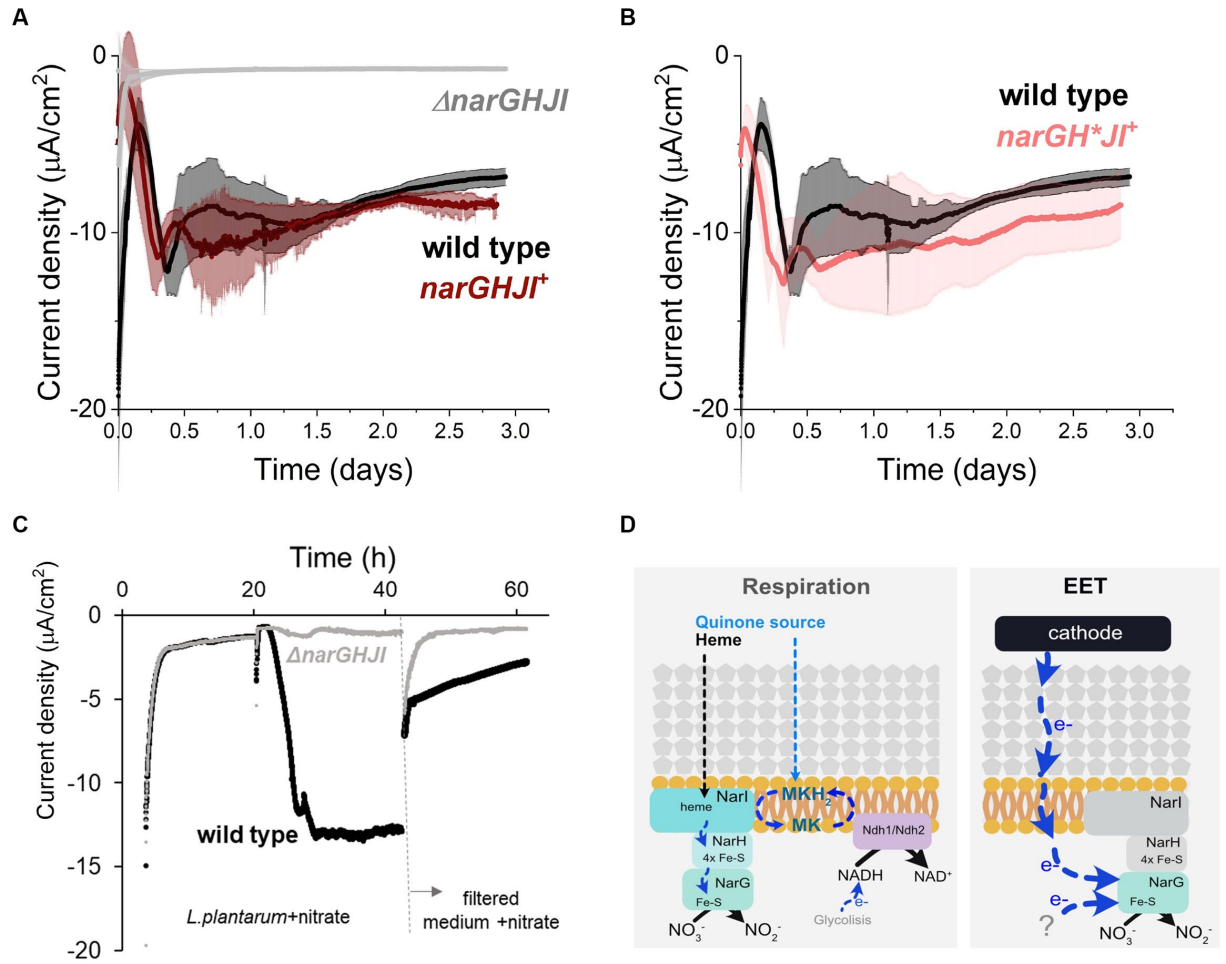
Role of nitrate reductase A in the cathodic current consumption. **(A)** Current density consumption of NCIMB8826 wild type strain compared to NCIMB8826 lacking the NarGHJI operon (Δ*narGHJI*) and the complemented strain with the operon in the Δ*narGHJI* background (*narGHJI^+^*). Comparison between wild type and Δ*narGHJI*, and between wild type and *narGHJI^+^* was performed in two independent assays. The plot shows current consumption of wild type in the second assay. **(B)** Current density consumption of NCIMB8826 wild type strain compared to NCIMB8826 expressing narGH*JI in the background of Δ*narGHJI* with Cys > Ala mutations in all the three [4Fe-4S] clusters of *narH*. **(C)** Current density over time of wild type and the mutant Δ*narGHJI* and current density of the filtered medium of the bioreactors (from 42 h) in a new BES. **(D)** Proposed mechanism of nitrate reduction and electron transport when electron donor (NADH from glycolysis) is in the cytoplasm (respiration) and proposed electron transport pathway when a cathode and a yet-unidentified molecules act as electron donors. For all experiments, nitrate (10 mM) and glucose (5 mM) were already present in the medium when the electrodes were polarized (ΔE_cathode_ = −600 mV_Ag/AgCl, sat. KCl_), and the error bars indicate the standard deviation from three replicates.

Since in our bioreactors EET from a cathode is nitrate-dependent, we next studied the role of the nitrate reductase A, the only enzyme known to catalyze nitrate reduction in *L. plantarum*. To this end, we tested the electron uptake capacity of a strain that lacks the operon Δ*narGHJI* encoding the sole nitrate reductase. We observed that current consumption and nitrate reduction were completely inhibited in the Δ*narGHJI* strain (Figure 5A; Supplementary Figure S7A). Furthermore, the catalytic redox center with a midpoint potential of −0.52 V that we previously observed in wild type strain (Supplementary Figure S1D) was absent from the voltammograms obtained using Δ*narGHJI* strain (Supplementary Figure S7B). Complementation of *narGHJI* via plasmid expression in the deletion background (*narGHJI*^+^ strain) restored the current consumption and nitrate reduction levels capacity to wild type levels (Figure 5A; Supplementary Figure S7C). Thus, nitrate reductase is essential for the nitrate reduction-dependent cathodic EET in *L. plantarum*.

To understand how electrons were transported across this enzyme during EET from a cathode, we analyzed the requirement of each subunit of nitrate reductase A in the process. This protein is membrane bounded and contains 3 subunits: NarI (subunit γ), NarH (subunit β), and NarG (subunit α) (Sparacino-Watkins et al., 2014; Figure 5D). When *L. plantarum* employs a soluble electron donor to perform anaerobic respiration on nitrate, both an external quinone and heme source are needed to restore NarI activity to receive electrons from a NADH-dehydrogenese (Brooijmans R. J. W. et al., 2009). Electrons are then transferred to NarH to the NarG subunit, the catalytic site of the enzyme (Figure 5D). Since *L. plantarum* is heme auxotroph and NarI is a bd-cytochrome described to accept electrons from membrane-embedded menaquinones (Guigliarelli et al., 1996), the transport of electrons cannot occur in the absence of exogenous heme and quinones. Thus, electrons from the cathode cannot be transported through NarI subunit. We then studied the electron transport across NarH and NarG during electron uptake from a cathode. These subunits harbor Fe-S clusters, described to be electron transport centers in nitrate reductase A in *L. plantarum* and other species (Guigliarelli et al., 1996; Sparacino-Watkins et al., 2014). We disrupted electron transport across narH by mutating specific cysteine residues within NarH that bind three 4Fe-4S to alanine, a mutation that results in the loss of several Fe-S clusters and full enzymatic activity (Guigliarelli et al., 1996). To build this mutant we constructed a plasmid to overexpress the narGH*JI genes in a Δ*narGHJI* background. Eliminating electron transport (Figure 5B) of NarH subunit did not affect current consumption capacity in *L. plantarum* nor nitrate reduction (Supplementary Figure S7C). This suggests that electrons do not flow through the Fe-S clusters of the NarH subunit when provided by a cathode. Thus, these results imply that electrons flow only via NarG subunit within the nitrate reductase when *L. plantarum* consumes electrons from a cathode.

Finally, since no redox-molecule was found to mediate electron uptake pin our previous tests, we next probed the possibility of *L. plantarum* employing an EET-mediated strategy to interact with the cathode by releasing detached proteins, like nitrate reductase, as previously observed for hydrogeneses and formate dehydrogenes in other species (Deutzmann et al., 2015). To do so, we performed a media swap assay to test current consumption capacity of cell-free medium (0.2 μm filter employed) from a current consuming bioreactor with cells and in the presence of nitrate (Figure 5C). This filtered medium could not support the current consumption levels observed when *L. plantarum* cells were present, indicating that free enzymes were not or minimally responsible for the electron uptake. We associate the background current with that one associated with abiotic nitrite reduction and a possible presence of oxygen traces. All together, these observations indicate that EET from a cathode involves the presence of whole cells with the nitrate reductase A, and NarG is the key enzyme for electron uptake. We therefore propose a model for this EET chain in *L. plantarum* in which a fraction of the electrons used to reduce nitrate flow from the cathode to the nitrate reductase A and, specifically, to NarG subunit through a yet unknown intermediate step(s) (Figure 5D).

## Discussion

Our results show *L. plantarum* can accept electrons from a cathode and couple this interaction to the reduction of the external electron acceptor nitrate. While *Lactobacillus* (Tejedor-Sanz et al., 2022), *Lactococcus* (Freguia et al., 2009) and *Enterococcus* (Keogh et al., 2018; Hederstedt et al., 2020) species are known to reduce extracellular electron acceptors, this is the first report of a LAB that can oxidize an extracellular electron donor like a cathode. Thus, *L. plantarum* is the first species discovered across the highly industrially relevant LAB group to possess an extracellular electron uptake capacity.

The rate of electron uptake was fastest when *L. plantarum* entered stationary phase and glucose was exhausted. Under this scenario, electron uptake correlated with a shift to heterofermentative metabolism and enhanced cell viability. Electron uptake in *L. plantarum* occurs with a dual reduction of both an endogenous (pyruvate) and an exogenous (nitrate) electron acceptor. A major difference observed between *L. plantarum* and other microorganisms capable of electrons uptake from cathodes is the nature of the observed metabolic shift. In contrast to species like *Clostridium* spp. (Choi et al., 2014; Kracke et al., 2016), electron uptake in *L. plantarum* does not stimulate NADH-consuming fermentation routes. This observation is intriguing because adding an electron donor is expected to increase the intracellular NADH/NAD^+^ ratio and thus cells would compensate by favoring NADH regenerating routes. A possible explanation of the metabolic shift observed could be that by favoring nitrate reduction (2 mol or 4 mol of NADH/mol of nitrate, when reduced to nitrite or ammonium, respectively) cells can more efficiently regenerate NADH than by reducing pyruvate to lactate or ethanol (1 or 2 mol of NADH/mol pyruvate, respectively). We hypothesize that this alternative mechanism of NADH regeneration through nitrate reduction may be beneficial in promoting ATP synthesis. This cathode-driven metabolic shift actually resembles those observed in *L. plantarum* under sugar limiting (Lindgren et al., 1990) and aerobic respiratory conditions (Zotta et al., 2017) if citrate or oxygen are present as electron acceptors, respectively. Those shifts all occur in stationary phase cells, and lactate is metabolized to acetate via pyruvate, possibly producing NADH, and also to CO_2_ and formate and succinate. In our study *L. plantarum* also produced succinate but formate was not detected when consuming electrons from a cathode. This hypothetical lactate-to-acetate conversion could be responsible for the higher acetate levels produced and cell viability observed under cathodic EET conditions. Further studies targeting key enzymes of *L. plantarum* involved in acetate production under anaerobic conditions are needed to elucidate the enzymatic mechanism behind the metabolic shift induced by the electron uptake.

Our results suggest that *L. plantarum* may perform a yet-unknown EET with a cathode, possibly direct or mediated through a self-synthesized molecule, since no exogenous free mediator usage (i.e., quinones, cysteine, flavins) could be detected, and media swap assays indicated whole cells are needed for significant current consumption. Electron uptake does not require the cofactors (heme, DHNA) or electron transfer proteins (CydABCD, Ndh2, PplA) needed by *L. plantarum* to reduce insoluble iron (III) or oxygen. In this EET machinery, nitrate reductase A is key for electron transport and catalysis. This enzyme is anchored to the cell membrane and most of its structure lies within the cytoplasm. Thus, we anticipate the existence of another factor channeling the electrons from the cathode to the nitrate reductase. Interestingly, the activity of this mechanisms depends upon the presence of a cathode and an exogenous electron acceptor. We discard the involvement of *c*-cytochromes (Khare et al., 2006; Rowe et al., 2021), hydrogeneses (Rowe et al., 2019) or a formate dehydrogenase (Deutzmann et al., 2015), components that are associated with electron uptake in other species, as *L. plantarum* does not possess genes encoding these proteins. Further studies are needed to elucidate the molecule or protein that ultimately is in direct contact with the cathode.

Why and how this novel EET capability is relevant under physiological conditions is enigmatic. This electron uptake capacity on *L. plantarum* could constitute a metabolic means for survival even without respiration. Many species like *L. plantarum* are cofactor auxotrophs. Therefore this extracellular electron uptake capacity could be correlated with a physiological advantage in niches deficient in those cofactors and containing extracellular electron donors like iron (II) (Kot et al., 1995) or a neighbor cell (Wang et al., 2016). Ferrous iron oxidation has been observed in the presence of some species of LAB (Kot et al., 1995, 1996), however the reaction was shown to be abiotically mediated by the cellular hydrogen peroxide produced. From a biotechnological perspective, the discovery of this pathway that electronically drives reduction reactions in *L. plantarum* and also a metabolic shift can have potential applications for bioelectrosynthesis. Overall, our advance can enable electricity-driven biosynthesis and biosensing in a wide-range of industrially relevant fermentative species, including species native of the human gut and oral cavity and Generally Regarded as Safe (GRAS) microorganisms.

## Methods

### Strains used and cultivation

*Lactiplantibacillus plantarum* NCIMB8826 strains were grown overnight (aprox. 18 h) at 37°C from glycerol stocks in MRS commercial media (BD, Franklin Lakes, NJ, USA) under static (non-aerated) conditions. Cells were harvested by centrifugation (5,250 g, 12 min, 4°C) and washed twice in 1xPBS. When *L. plantarum* wild type EET activity versus the mutants was compared, cells were grown as described and the number of cells was normalized across the strains prior to its inoculation in the electrochemical cells. For growing the strains in the bioelectrochemical reactors we used a Chemically Defined Media (CDM) (Aumiller et al., 2023) (see composition in SI) containing 5 mM of glucose and 10 mM of nitrate. The media was adjusted to pH 6.5 and then filter sterilized. For the tests for studying growth of *L. plantarum*, the strain containing the empty vector pSIP403, and the CDM also contained erythromycin (10 μg/mL). The complementation strains of *narGHJI* and the narGH*JI mutated strains were grown with 10 μg/mL erythromycin.

### Strains construction

The strains, plasmids, primers and DNA fragments used in this study are listed in Supplementary Tables S2–S4, respectively. *L. plantarum* NCIMB8826 wild type, *pplA*, *ndh2* and *narGHJI* strains were kindly provided to us by Maria Marco group at the UC Davis. *L. plantarum* NCIMB8826 *eetA/B* deletion mutant was constructed by CRISPR-Cas9 toolbox according to Huang et al. (2019). Briefly, the upstream and downstream homologous arms were amplified from genomic DNA of *L. plantarum* (see Supplementary Table S4 for primers). The crRNA targeting *eetA/B* was designed by using CRISPOR.^1^ The sgRNA was synthesized as an oligo to insert into the pHSP02 backbone. All the fragments were assembled in ApaI-XbaI digested pHSP02 editing plasmid to create pSTS02 by Gibson assembly (Gibson et al., 2009). For CRISPR editing, *L. plantarum* strain harboring helper plasmid pLH01 was induced with 100 ng/mL Sakain P peptide (GenScript, Piscataway, NJ) for RecE/T expression and was subsequently prepared as competent cells. The pSTS02 was then delivered into *L. plantarum* NCIMB8826 by electroporation. The deletion mutants were selected on MRS plates containing 10 μg/mL erythromycin and 10 μg/mL chloramphenicol. Colony PCR was performed to confirm the deletion of the target gene (see Supplementary Table S4 for primers).

The complementation of *narGHJI* was achieved by using pSIP403 plasmid (Sørvig et al., 2003). The *narGHJI* gene fragments were amplified from genomic DNA of *L. plantarum* NCIMB8826 (see Supplementary Table S4 for primers) and was cloned into NcoI-EcoRI digested pSIP403 backbone by Gibson assembly (Gibson et al., 2009) to generate pSL02. The *narH* [4Fe-4S] Cys > Ala mutation was constructed based on pSL02. Gene fragment containing the Cys > Ala mutations was synthesized as a gBlock (Twist Bioscience, San Francisco, CA) and was inserted into pSL02 by Gibson assembly to create pSL06. All plasmids were verified by Sanger sequencing and were delivered in *L. plantarum* NCIMB8826 by electroporation.

### Bioelectrochemical analyses

The bioreactors consisted of double chamber electrochemical cells (Adams & Chittenden, Berkeley, CA) with a cation exchange membrane (CMI-7000, Membranes International, Ringwood, NJ) that separated them. We used a 3-electrode configuration consisting of an Ag/AgCl sat KCl reference electrode (BASI), a titanium wire counter electrode, and a working electrode of either 6.35-mm-thick graphite felt working electrode of 4×4 cm (Alfa Aesar) or a graphite rod of 1.2 cm of diameter and 3 cm length (used for cell viability tests), both with a piece of Ti wire threaded as a current collector and connection to the potentiostat. We used a Bio-Logic Science Instruments potentiostat model VSP-300 for performing the electrochemical measurements. The bioreactors were sterilized by filling them with ddH_2_O and autoclaving at 121°C for 30 min. After this, each chamber media was replaced with 150 mL of filter sterilized CDM media (for the working electrode chamber), and 150 mL of M9 media (BD) (for the counter electrode chamber). When indicated, nitrate (aprox. 10 mM) was supplemented as electron acceptor and glucose as carbon source (5 or 10 mM). When appropriate, the CDM was supplemented (before filter-sterilized) with 20 ug/mL of DHNA diluted 1:1 in DMSO: ddH_2_O where appropriate or with heme at a final concentration of 10 ug/mL (diluted 1:1 in DMSO: ddH_2_O). Riboflavin was omitted from the mineral stock solution of the CDM in the test in which the effect of the presence of flavins in the medium was studied.

The media in the working electrode chamber was mixed with a magnetic stir bar for the course of the experiment and N_2_ gas was continuously purged in the working electrode chamber to maintain anaerobic conditions. The applied potential to the working electrode was of −0.6 V versus Ag/AgCl (sat. KCl) (BASI). All the experiments were tested under 30°C. Reactors at open circuit (OC) were similarly assembled and used as control for non-current circulating conditions. Once the current stabilized, the electrochemical cells were inoculated to a final OD_600nm_ of 0.1–0.15 with the cell suspensions prepared in M9 medium. Current densities are reported as a function of the geometric surface area of the electrode (16 cm^2^ for carbon felt). Differential Pulse Analyses tests were performed from −0.75 V to 0.4 V, 50 mV pulses height, 500 ms pulses width, 1 mV step height and 1,000 ms step time. In the experiment where metabolites production was tested, we inoculated with an initial OD_600NM_ = 0.05 to slow the consumption of glucose and examine metabolites production within time.

The bioreactors were sampled under sterilized conditions at different time points for subsequent analysis. The samples for organic acids analyses were centrifuged (15,228 g for 7 min) and the supernatant was separated for HPLC assessments.

### Media swap assay

Bioreactors were prepared using the same methodology as before, with the CDM medium containing 5 mM glucose and 10 mM of nitrate. We used an erytromycin resistant strain (10 ug/mL) as electron uptaking strain and Δ*narGHJI* as control. We cultured the strains in a BES with the cathode polarized at ΔE_cathode_ = −600 mV_Ag/AgCl, sat. KCl, and after aprox._ 42 h, we collected and filtered the medium (0.22 μm) from each BES, and used add it all into a new abiotic BES to measure current consumption in the absence of cells.

### Analysis of organic acids

Organic acids, ethanol and sugar concentrations were measured by HPLC (Agilent, 1,260 Infinity), using a standard analytical system (Shimadzu, Kyoto, Japan) equipped with an Aminex Organic Acid Analysis column (Bio-Rad, HPX-87H 300 × 7.8 mm) heated at 60°C. The eluent was 5 mM of sulfuric acid, used at a flow rate of 0.6 mL min^−1^. We used a refractive index detector 1,260 Infinity II RID. A five-point calibration curve based on peak area was generated and used to calculate concentrations in the unknown samples. We included the following standards in the HPLC measurements: acetate, formate, pyruvate, malate, lactate, succinate, oxalacetate, fumarate, ethanol, acetoin, butanediol, mannitol and glucose. No gaseous products were measured.

### Measurement of biomass and growth in the bioreactors

Bioreactors were shaken to remove the cells attached to the working electrode and afterwards sampled to measure total biomass (dry weight). Dry weight was determined after taking 75 mL of sample of the bioreactor supernatant after the current consumption was significantly reduced. Pellets were harvested by centrifugation (5,250 g, 12 min, 4°C) and washed twice in ddH2O (50 mL/sample/wash) to remove the salts. Afterwards the suspended pellets were transferred to 1.5 mL Eppendorf tubes, centrifuged again and let dry at 30°C using an evaporator. The microfuge tubes were then cooled in a desiccator for 30 min and the weight of each tube was measured to determine cell weight. The difference between the weight of each tube with the pellet and before containing it allowed us to determine the dry weight mL^−1^. Samples for CFU enumeration were collected under sterile conditions at the time of inoculation and at the time of approximately maximum current density. Samples were serially diluted (1:1,000 to 1:10,00,000) in sterile PBS, and plated on MRS for CFUs enumeration after overnight incubation at 30°C.

### Analysis of nitrate and nitrite

Nitrate and nitrite were measured by either ion chromatography and/or with an enzymatic kit. The chromatograph used was a Dionex ICS-2100 system (Thermo Scientific, USA). The enzymatic kit used was a Cell Biolabs’ OxiSelect™ Nitric Oxide (Nitrite/Nitrate) Assay Kit (Cell Biolabs, USA). Nitrate in the samples was converted to nitrite by nitrate reductase enzyme and then total nitrite was detected with Griess reagents as a dye product.

### Calculations of electron, NADH, and ATP balances

The coulombic efficiency or electron recovery on the cathode was calculated as the fraction of the electrons consumed on the cathode over the total theoretical electrons needed to reduce nitrate to nitrite. The electrons consumed on the cathode were estimated by integrating the area (charge) under the chronoamperometric curve [current response (A) over time (s)]. This obtained charge was then converted to mol of electrons using the Faraday constant (96,485.3 A*s mol^−1^ electrons) to obtain the experimental electron consumption. We estimated the total theoretical electrons used to reduce all the nitrate measured to nitrite considering that 2 mol of electrons are needed to reduce 1 mol of nitrate.

Total NADH produced was calculated based on the known stoichiometry of the fermentation pathways to assume 1 mol of NADH is produced for each mole of lactate, acetate, ethanol, pyruvate, succinate and formate detected from glucose fermentation. For EET pathways, the concentration of additional NADH generated from the electron supplies by the cathode was calculated by determining the moles of electrons supplied by the cathode, as previously described (Tejedor-Sanz et al., 2020). Under OC conditions, all NADH was generated from fermentation, while under EET conditions, NADH was generated from fermentation and from the cathode electron supply. *L. plantarum* can consume NADH in three ways: by reducing pyruvate to lactate (1 mol NADH/mol lactate produced) or to ethanol (2 mol NADH/mol ethanol produced), by reducing pyruvate or phosphoenolpyruvate to succinate via the reduced branch of the tricarboxylic acid cycle (2 mol NADH/mol succinate produced), and by reducing nitrate to nitrite (1 mol of NADH/mol nitrate consumed).

## Data availability statement

The raw data supporting the conclusions of this article will be made available by the authors, without undue reservation.

## Author contributions

ST-S: Conceptualization, Data curation, Formal analysis, Investigation, Methodology, Supervision, Visualization, Writing – original draft, Writing – review & editing. SL: Data curation, Formal analysis, Investigation, Methodology, Visualization, Writing – review & editing. BBK: Data curation, Formal analysis, Investigation, Methodology, Validation, Writing – review & editing. CA-F: Conceptualization, Funding acquisition, Methodology, Project administration, Resources, Supervision, Writing – review & editing.

## Footnotes

^1^ http://crispor.tefor.net/
