# Supplementary material for: Extracellular electron uptake from a cathode by the lactic acid bacterium Lactiplantibacillus plantarum
Source: Front Microbiol. 2023 Nov 23;14:1298023. doi: 10.3389/fmicb.2023.1298023 (PMC10701730; doi:10.3389/fmicb.2023.1298023)
Supplement: Supplementary file 1 [file Data_Sheet_1.pdf]

Supplementary Information

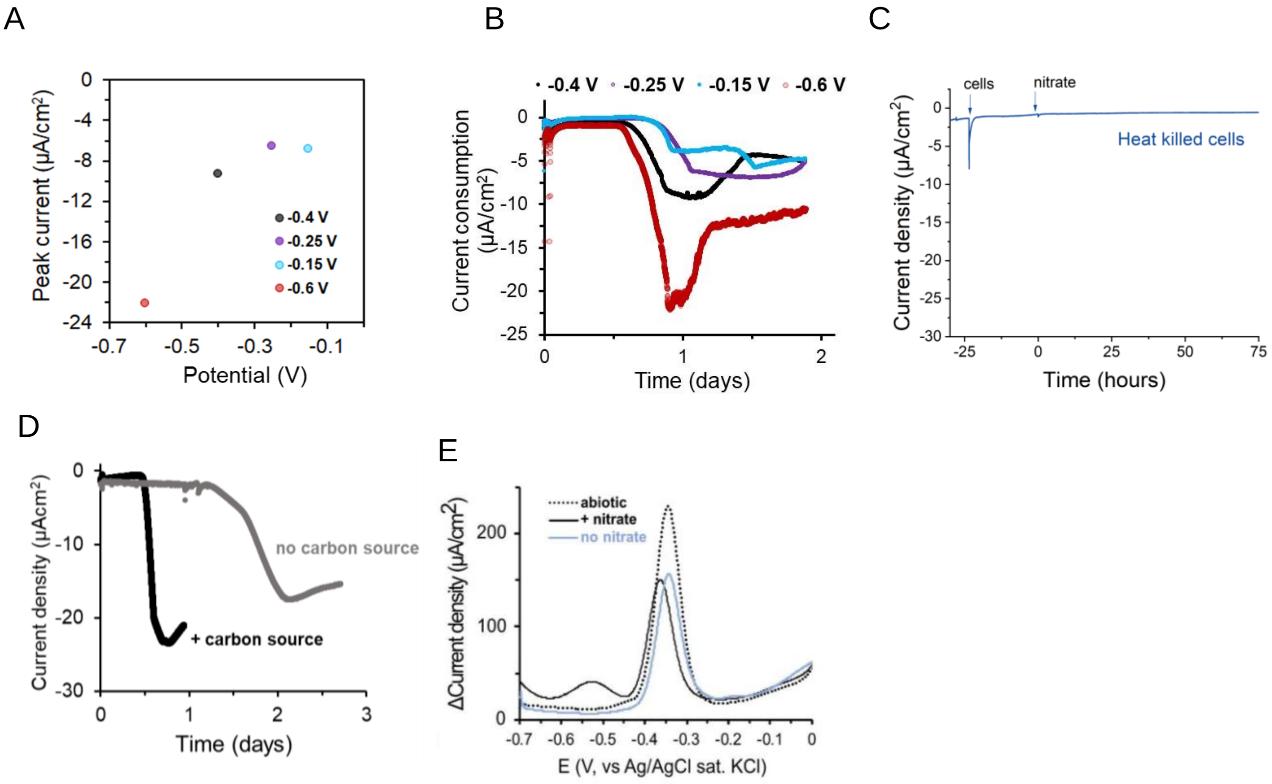

Supplementary Figure S1: Extracellular electron uptake in *L. plantarum* under different conditions. A) Peak current consumed and B) current density over time, by *L. plantarum* at different cathode potentials (vs Ag/AgCl, sat. KCl) with 5 mM of glucose in the medium and with 10 mM of nitrate. C) Abiotic current consumed by heat killed cells of *L. plantarum* upon the addition of nitrate (11.2 mM) with a cathode polarized to ΔE = -600 $mV_{Ag/AgCl,\ sat.\ KCl}$ with 5 mM of glucose in the medium. D) Current consumption in the presence and absence of a carbon source (5 mM of glucose) in the medium and with 10 mM of nitrate. The electrodes were polarized to ΔE = -600 $mV_{Ag/AgCl,\ sat.\ KCl}$. E) Differential pulse voltammetry performed in the presence and absence of nitrate (10 mM) with a polarized cathode (ΔE=-600 $mV_{Ag/AgCl,\ sat.\ KCl}$) and under abiotic conditions. For the assays shown in D) and E), nitrate and glucose were already present in the medium at t=0, when appropriate. The tests in C, D and E were performed in duplicate bioelectrochemical reactors.

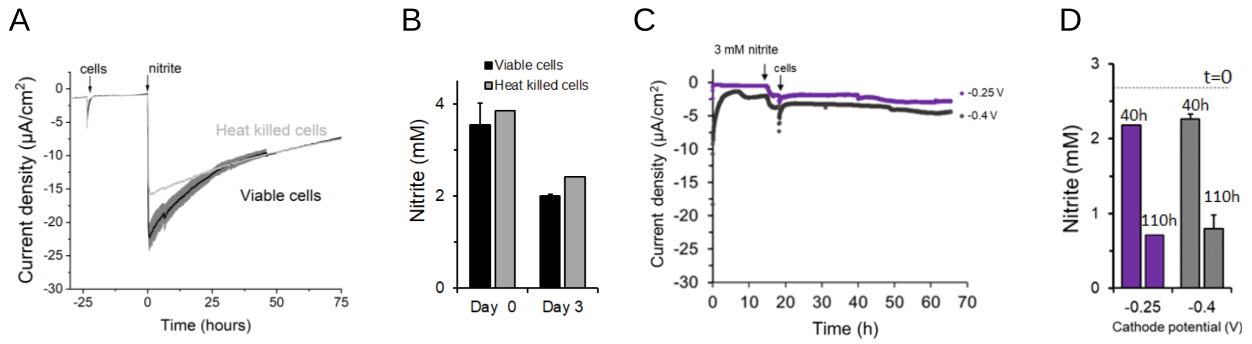

Supplementary Figure S2: Biotic and abiotic reduction of nitrite. A) Biotic and abiotic current consumption by heat killed cells of *L. plantarum* upon the addition of nitrite (3.7 mM) with a cathode polarized to ΔE = -600 $mV_{Ag/AgCl, sat. KCl}$ and with 5 mM of glucose in the medium. B) Nitrite levels at the time of nitrite addition to the bioreactors and after 3 days in the presence of viable cells and heat killed cells of *L. plantarum* with a cathode polarized to -ΔE = -600 $mV_{Ag/AgCl, sat. KCl}$. C) Current consumption of *L. plantarum* upon the addition of nitrite (3 mM) with a cathode polarized to ΔE = -250 and -400 $mV_{Ag/AgCl, sat. KCl}$ with 5 mM of glucose in the medium. The arrows indicate nitrite addition to the medium (black arrow) and cell additions to the medium (arrow). D) Nitrite levels in the bioreactors polarized to ΔE = -250 and -400 $mV_{Ag/AgCl, sat. KCl}$ at time zero and after 40 and 110h of being polarized and cells added.

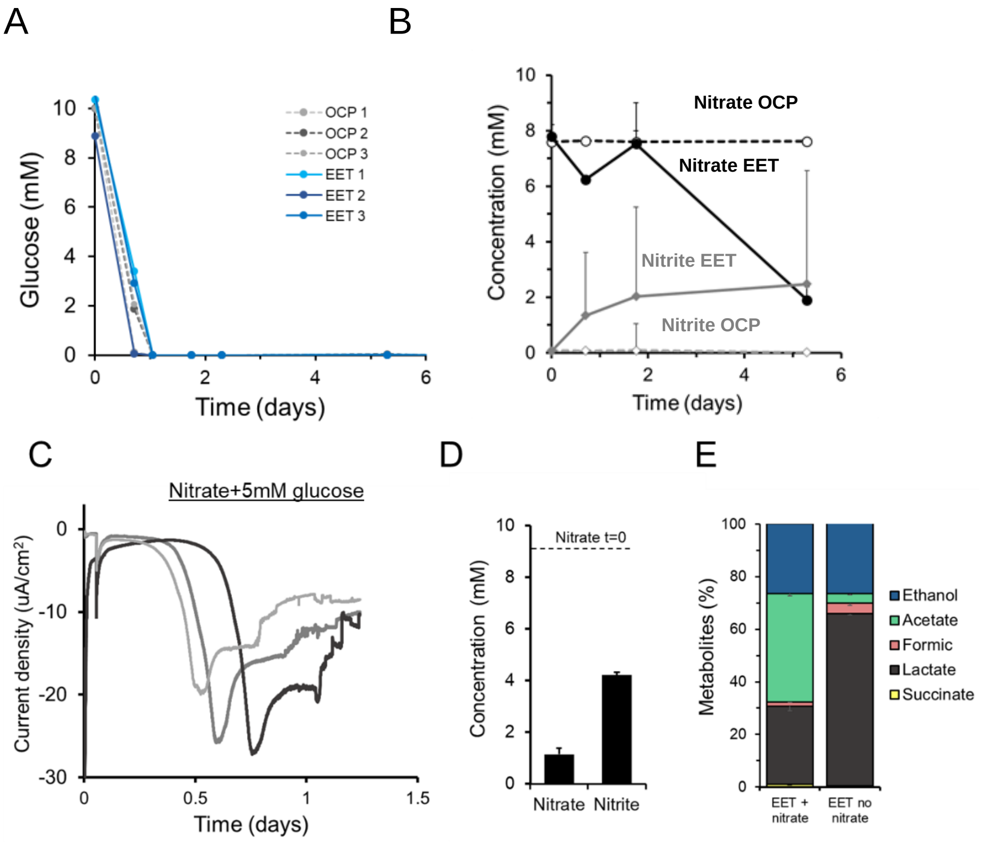

Supplementary Figure S3: A) Glucose levels in the bioelectrochemical reactors for each

biological replicate under EET and OCP conditions. B) Nitrate and nitrite reduction in the bioelectrochemical reactors. C, D and E) Impact of EET in the presence of 5mM of glucose and nitrate in the medium. Panel B shows current density consumption when nitrate is present of each individual replicate. Panel D and E show nitrate and nitrite levels and metabolites distribution, respectively, by day 1.25 of the 2 conditioned tested. The electrodes were polarized to ΔE = -600 $mV_{Ag/AgCl,\ sat.\ KCl}$, and the error bars indicate the standard deviation from three biological replicates.

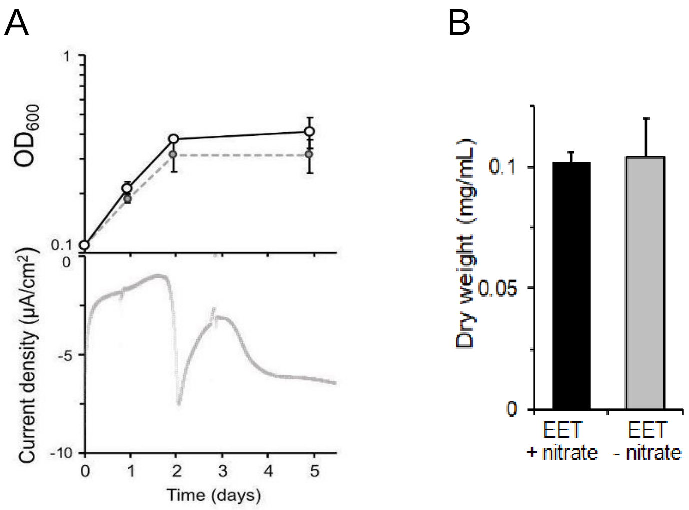

Supplementary Figure S4: A) Growth (OD_{600nm}) under electron uptake conditions (black line) and in the presence of an electrode maintained at OC (dotted grey line). Nitrate was supplied as an electron acceptor in the medium. Evolution of current density of one of the bioreactors is shown (gray dots, bottom panel). B) Dry weight under EET conditions in the presence and absence of nitrate and with glucose as carbon source. The electrodes were polarized to ΔE = -600 $mV_{Ag/AgCl,\ sat.\ KCl}$, and the error bars indicate the standard deviation from three biological replicates.

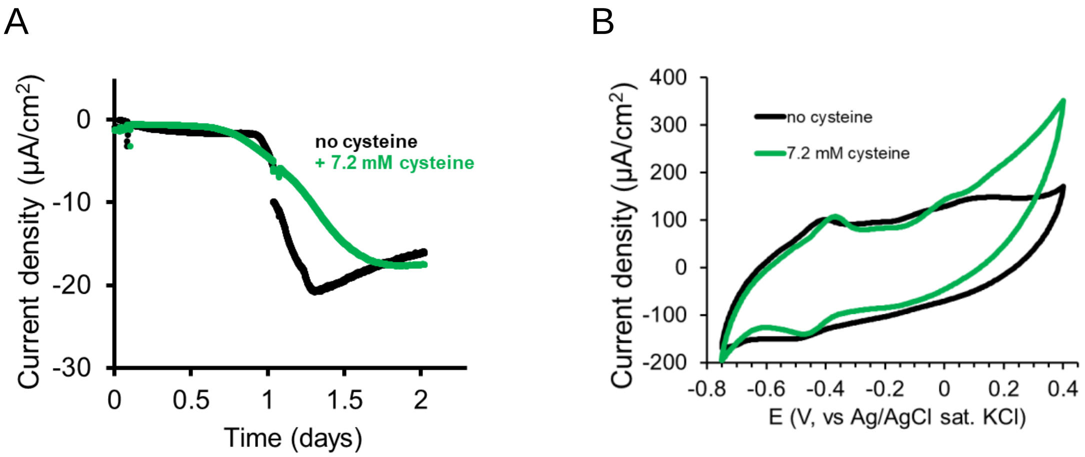

Supplementary Figure S5: Cysteine impact on cathodic EET. A) Current consumption and

B) cyclic voltammetry analysis (scan rate of 5 m/Vs) by *L. plantarum* in the presence of glucose (5 mM) and nitrate (10 mM) and a polarized cathode (ΔE = -600 $_{mVAg/AgCl,\ sat.\ KCl}$) in the presence and absence of cystine (7.2 mM) in the medium.

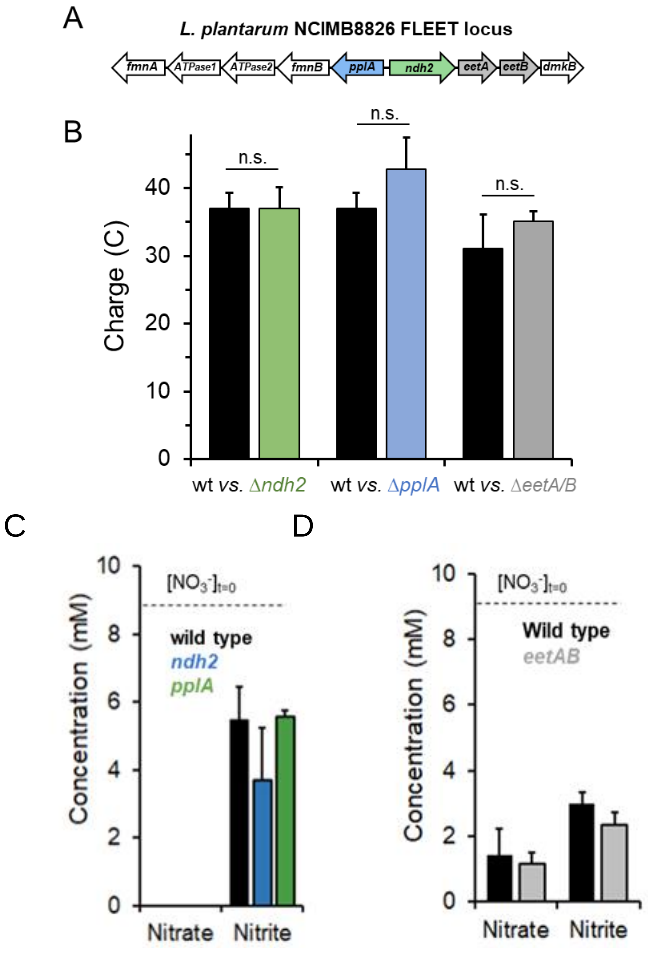

Figure S6: Role of anodic EET genes of *L. plantarum* in the extracellular electron uptake from a cathode. A) Genes within the FLEET locus that confer *L. plantarum* extracellular electron capacity ability with anodes. B) Total electron uptake quantified as charge consumed on the cathode by *L. plantarum* NCIMB8826 wild type strain compared Δndh2, ΔpplA and ΔeetA/B strains. Charge was calculated from current consumption over a period of 3 days of electron uptake. Assays to compare charge consumed by wild type vs. Δndh2 and ΔpplA were performed using the same culture of wild type strain, whereas a different culture was used to test ΔeetA/B electron uptake. C) and D) Nitrate and nitrite levels measured in the bioelectrochemical reactors after aprox. 4 days after inoculation of the bioreactors operated with wild type or mutant strains tested in the presence of a cathode (-600 mV vs Ag/AgCl vs sat. KCl), and glucose (5 mM) as carbon source. For all experiments, nitrate was already present in the medium when the electrodes were polarized and the error bars indicate the standard deviation from three biological replicates.

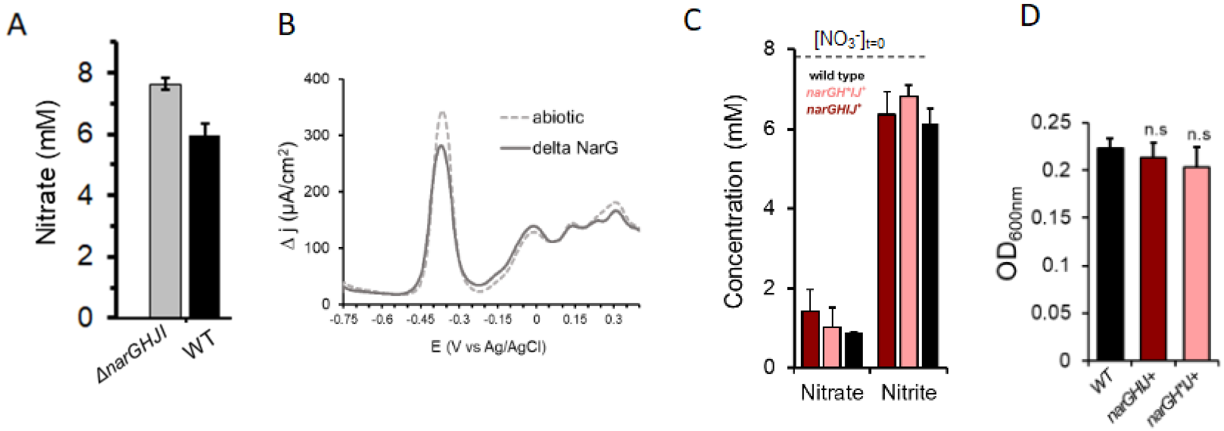

Supplementary Figure S7: Nitrate reductase subunit G role in extracellular electron uptake from a cathode and nitrate reduction. A) Residual nitrate at the point of peak current consumption in the bioreactors inoculated with wild type and Δ*narGHJI* strains. B) Differential pulse voltammetry curves performed on the cathode previously polarized to -600 mV (vs Ag/AgCl vs sat. KCl), under EET conditions (with glucose and nitrate) with *ΔnarG* and under abiotic conditions. C) Residual nitrate and nitrite levels measured in the bioelectrochemical reactors after aprox. 3 days after inoculation of the different strains tested in the presence of a cathode (-600 mV vs Ag/AgCl vs sat. KCl), and glucose (5 mM) as carbon source. D) Growth of wild type strain compared to the nitrate reductase mutants in the bioelectrochemical reactors after aprox. 3 days after inoculation of the different strains tested in the presence of a cathode, and glucose (5 mM) as a carbon source. For all experiments, nitrate was already present in the medium when the electrodes were polarized and the error bars indicate the standard deviation from three (plots C and D) or two (plot A) biological replicates.

Supplementary Table S1. Chemically defined medium recipe.

**CDM component**	**Final Concentration (g/L)**
**Buffers and salts**	
MOPS (3-(N-morpholino)propanesulfonic acid)	8.371
K_2HPO_4	0.871
NH_4Cl	1.070
Na_2SO_4	1.420
**Metals**	
$MgCl_2$ * $6H_2O$	0.203
$MnCl_2$ * $4H_2O$	0.001
$FeSO_4$ * $7H_2O$	0.014
**Amino acids**	
Casamino acids	3.000
Cysteine-HCl * H_2O	0.145
Tryptophan	0.050
**Wolfe's Vitamins**	

Pyridoxine HCl	0.002
Thiamine HCl	0.001
Riboflavin	0.001
Nicotinic acid	0.001
Calcium D-(+)-pantothenate	0.001
p-Aminobenzoic acid	0.001
Thioctic acid (α-Lipoic acid)	0.001
Biotin	0.0004
Folic acid	0.0004
Vitamin B12	0.00002
**Wolfe's Minerals**	
Nitrilotriacetic acid (NTA)	0.3
$MgSO_4 * 7H_2O$	0.6
$MnSO_4 * H_2O$	0.1
NaCl	0.2
$FeSO_4 * 7H_2O$	0.02
$CoCl_2 * 6H_2O$	0.02
$CaCl_2$	0.02
$ZnSO_4 * 7H_2O$	0.02
$CuSO_4 * 5H_2O$	0.002
$AlK(SO)_4 * 12H_2O$	0.002
H_2BO_3	0.002
$Na_2MoO_4 * 2H_2O$	0.002

Supplementary Table S2. Strains used in this study.

Strains	Description	Genotype	Reference
L. plantarum NCIMB8826	wild type strain	NCIMB8826	(Tejedor Sanz et al, 2022)
L. plantarum Δndh2	Deletion mutant of NCIMB8826 lacking *ndh2*	NCIMB8826 *Δndh2*	(Tejedor Sanz et al, 2022)
L. plantarum ΔpplA	Deletion mutant of NCIMB8826 lacking *pplA*	NCIMB8826 *ΔpplA*	(Tejedor Sanz et al, 2022)
L. plantarum ΔnarGHJI	Deletion mutant of NCIMB8826 lacking the operon *narGHIJ*	NCIMB8826 *ΔnarGHJI*	(Tejedor Sanz et al, 2022)
L. plantarum RCL004	NCIMB8826 harboring pSIP403 empty vector	NCIMB8826+ PSIP 403	This study
L. plantarum	NCIMB8826 expressing	NCIMB8826	This study

RCL005	*narGHJI* in the background of *ΔnarGHJI*	*ΔnarGHJI* + pSL02	
L. plantarum RCL006	NCIMB8826 expressing *narGH*JI* in the background of *ΔnarGHJI* with Cys>Ala mutations in all the three [4Fe-4S] clusters of *narH*	NCIMB8826 *ΔnarGHJI* + pSL06	This study
L. plantarum RCL008	Deletion mutant of NCIMB8826 lacking *eetA/B*	NCIMB8826 *ΔeetA/B*	This study
L. plantarum SL076	NCIMB8826 ΔnarGHJI + pSIP403 empty vector	NCIMB8826 *ΔnarGHJI* +PSIP403	This study

Supplementary Table S3. Plasmids used in this study.

**Plasmids**	**Description**	**Reference**
pSIP403	Sakacin P inducible plasmid for gene expression in *L. plantarum.*	(Sørvig *et al*. 2003)
pLH01	Helper plasmid for CRISPR-Cas9 toolbox containing RecE/T recombinase.	(Huang *et al*. 2019)
pHSP02	Editing plasmid for CRISPR-Cas9 toolbox containing Cas9 nuclease, P11-sgRNA, and homologous arms.	(Huang *et al*. 2019)
pSTS02	Editing plasmid for *eetA/B* deletion in *L. plantarum*.	This study
pSL02	Sakacin P induced expression of *narGHJI.*	This study
pSL06	Sakacin P induced expression of *narGH*JI* with Cys>Ala mutations in all the three [4Fe-4S] clusters of *narH.*	This study

Supplementary Table S4. Primers and gBlocks used in this study.

Primer name	Sequence (5'-3')	Use
ΔeetA/B up_arm-F	gtggcaccgagtcggtgcttttttgagtaagaacgttgaccagactgac	Upstream homologous arm for *eetA/B* deletion

ΔeetA/B up_arm-R	tgtgttgccgattgcataaggttaaccctccaattcaatgaaat	Upstream homologous arm for *eetA/B* deletion
ΔeetA/B down_arm-F	cttatgcaatcggcaacacaat	Downstream homologous arm for *eetA/B* deletion
ΔeetA/B down_arm-R	gatcacatctttttctaaactagggcccttaattatgccggtccaacagc	Downstream homologous arm for *eetA/B* deletion
ΔeetA/B sgRNA	tggacatactatgatatattctaga**tgaaaccacgtataacgccg**gttttagagctagaaatagcaagttaaaataaggctagtccgttatcaacttgaaaaagtggcaccgagtcggtg	DNA fragment containing sgRNA sequence targeting *eetA/B (***bold text** indicates 20nt crRNA*)*
sgRNA-F	tggacatactatgatatattctag	Amplify sgRNA fragment
sgRNA-R	caccgactcggtgc	Amplify sgRNA fragment
ΔeetA/B check-F	acccgctagatgcgttataattaa	Check *eetA/B* deletion
ΔeetA/B check-R	agattatgacctgttgaatcggg	Check *eetA/B* deletion
narGHJI-F	ataggagtatgattcccatgaaaaaatcccgtttctttaaaaatgtcgaaaagt	Amplify *narGHJI* from *L. plantarum*'s genome
narGHJI-R	accgaattcctcgagtctagctaaactcgtggccgccgcc	Amplify *narGHJI* from *L. plantarum*'s genome
*narGH*JI*_frag_1-R	accccgattacccgtaatcgtt	NarH Cys>Ala mutant fragment_1
*narGH*JI*_frag_3-F	tgagggccgcgattcgat	NarH Cys>Ala mutant fragment_3
gBlock name	Sequence (5'-3')	Use
*narGH*JI*_frag 2	gaaatccaagaaccgttgtcaacgattacgggtaatcggggtggctcgcacaatgcgccaacccaaattcatgtgaaaccaacccaaatggtcggtggttatggtcagttgagttatggtttcaattattatggcccaatcggtaatcaacgggacctgtacgtgaacgtacggaagttaaagaaggtgaactggaatgaagattaaagcacaaattgggatggtcttaaaccttgataag**GCT**attggt**GCT**catacc**GCT**tcggtcact**GCT**aaaaatacttggaccaatcgtcctggggcagaatacatgtggtttaacaatgtcga	NarH Cys>Ala mutant fragment_2 (gray shadows indicate [4Fe-4S] clusters, **uppercase bold texts** indicate Cys>Ala mutation sites)

	gaccaaacccggtgtcggctacccgaaacgctgggaagat<br>gaagatcactataaaggtggttgggagttgaacagtaaaggt<br>aaacttcaactccgagcaggtaataaggtcaataagatcgca<br>ctcggaaagatttttatcaacctgatatgccagaattggacga<br>ctattatgaaccatggacgtatgattaccaaaccttattcggac<br>ctgaaaaggcccatcaaccagttgcacgcgcgacgtcgca<br>gattactggattaaagatggatcttaagacgggccccaactg<br>ggatgatgatttagcaggatcacctgaatatttcaaagcagac<br>ccgaacatggaaaagatagagactgatatcaaagcgaacttt<br>gaacaggccttcatgatgtacctcccacgtcta**GCT**gaac<br>at**GCT**ttgaacgcacca**GCT**gtggcgtca**GCT**cctt<br>ctggggcgatgtataagcgtgatgaagacggtatcgtcttgg<br>ttgatcaagaacgg**GCT**cgcggctggcgtttt**GCT**atg<br>acgggt**GCT**ccttataagaaggtctacttcaactggaaaa<br>cccataaggctgaaaaatgcacgttctgttatccacggatcg<br>aagaaggtgaaccgactgtctgtgccgagacttgtgtcggtc<br>ggatccggtatatcggcgccatgttatatgacgctgatcgggt<br>cgaagaagcggccgaaacgcctgaggaagaccaattatac<br>caagcacagttagatttgttcttggaccccaatgatccagaca<br>tcattgaacaagcgttggcggatggcatctctgaagaaatgtt<br>ggaagctgctcagaattcgccgatttaccggatggccgtcga<br>agaaaaagatcgcttttccgcttcatcctgaatatcggacgatg<br>cccatggtttggtacatcccaccattgtcaccagtcatgaatta<br>ttttgagggccgcgattcgatcaaagatcctgagatgattttcc	

Estimation of nitrite reduction in the electrochemical cells

Initial experiments of heat-killed cells in bioreactors indicated that nitrite reduction occurred under both biotic and abiotic conditions at -0.60 V (Supplementary Fig. 2A and 2B). To minimize the abiotic reduction of nitrite, we polarized the cathode to more positive potentials (-0.25 V and -0.40 V) and again probed nitrite reduction under both biotic and abiotic conditions (Supplementary Fig. 2C and Fig. 2D). From this data, we estimated that nitrite reduction coupled to electron uptake by L. plantarum was minimal (<2.5%), and that instead, most of this reduction (>91%) was catalyzed by *L. plantarum*, but without use of the cathode as electron donor
.

Analysis of electrochemical (abiotic) and bioelectrochemical nitrite reduction on the cathode.

	**POTENTIAL CATHODE (v VS Ag/AgCl sat. KCl)**	
	**-0.25**	**-0.40**
**Charge (C)**	-5.25	-4.98
**mmol e**	-0.05	-0.05

**Experimental nitrite reduced (mM)**	2.04	1.96
**mM nitrite reduced abiotically with cathode**	0.14	0.12
**mM nitrite reduced with cathode**	0.18	0.17
**% nitrite reduced abiotically with cathode**	6.95	6.31
**% total nitrite reduced with cathode**	8.87	8.78
**% nitrite reduced biologically with cathode**	1.92	2.47
**% nitrite reduced without the cathode (%)**	91.13	91.22

Results of the electron balances suggest that aprox. 91 % of the nitrite reduced in the bioelectrochemical reactors at -0.25 V and -0.4 V does not proceed with the cathode as electron donor. Our calculations show that, if taken the fraction of nitrite reduced purely electrochemically with the cathode, only a 1.9% (at -0.25 V) and 2.5% (at -0.4 V) would be reduced by *L. plantarum* using the cathode as electron donor.
